# Identification of conclusive association entities in biomedical articles

**DOI:** 10.1186/s13326-018-0194-9

**Published:** 2019-01-07

**Authors:** Rey-Long Liu

**Affiliations:** Department of Medical Informatics, Tzu Chi University, Hualien, Taiwan, Republic of China

**Keywords:** Conclusive association entity, Statistical indicator, Visualization, Exploratory analysis

## Abstract

**Background:**

*Conclusive association entities* (CAEs) in a biomedical article *a* are those biomedical entities (e.g., genes, diseases, and chemicals) that are specifically involved in the associations concluded in *a*. Identification of CAEs among candidate entities in the title and the abstract of an article is essential for curation and exploration of conclusive findings in biomedical literature. However, the identification is challenging, as it is difficult to conduct semantic analysis to determine whether an entity is a *specific* target on which the reported findings are *conclusive* enough.

**Results:**

We investigate how five types of statistical indicators can contribute to prioritizing the candidate entities so that CAEs can be ranked on the top for exploratory analysis. The indicators work on titles and abstracts of articles. They are evaluated by the CAEs designated by biomedical experts to curate entity associations concluded in articles. The indicators have significantly different performance in ranking the CAEs identified by the biomedical experts. Some indicators do not perform well in CAE identification, even though they were used in many techniques for article retrieval and keyword extraction. Learning-based fusion of certain indicators can further improve performance. Most of the articles have at least one of their CAEs successfully ranked at top-2 positions. The CAEs can be visualized to support exploratory analysis of conclusive results on the CAEs.

**Conclusion:**

With proper fusion of the statistical indicators, CAEs in biomedical articles can be identified for exploratory analysis. The results are essential for the indexing of biomedical articles to support validation of highly related conclusive findings in biomedical literature.

**Electronic supplementary material:**

The online version of this article (10.1186/s13326-018-0194-9) contains supplementary material, which is available to authorized users.

## Introduction

*Conclusive association entities* (CAEs) in a biomedical article *a* are those biomedical entities (e.g., genes, diseases, and chemicals) that are specifically involved in the associations concluded in *a*. Consider the article in Table 1 as an example (ID in the search engine PubMed is 6,492,995). The article is curated by CTD (Comparative Toxicogenomics Database), which maintains a database of associations between chemicals, genes, and diseases [1]. An association is curated only if CTD scientists verify that conclusive evidences are reported to support the association. The article mentions seven entities in the set of entities considered by CTD. With this article, several associations are curated: the gene prolactin interacts with two chemicals 2-bromolisuride and lisuride; while the disease hyperprolactinaemia has a ‘marker’ association with two chemicals 2-bromolisuride and reserpine, as well as a ‘therapeutic’ association with the chemical lisuride. These chemicals as well as the gene and the disease can thus be CAEs of the article. Other entities in the article are non-CAEs: Dopamine is not a specific target on which the conclusions are made, while transdihydrolisuride is an entity on which the reported findings may not be conclusive enough (as its effects may change in different conditions).

**Table 1:**
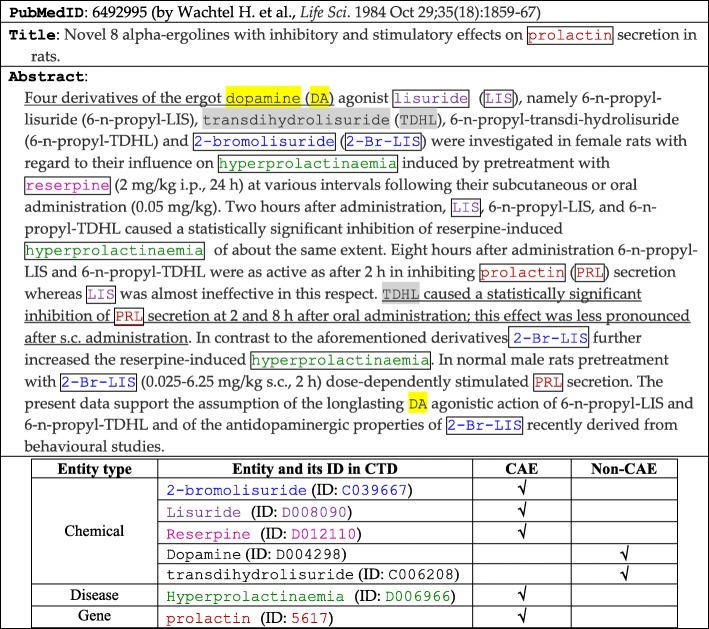
An article curated by CTD scientists. Five entities are identified as CAEs (see the boxed entities), which are the ones on which conclusive associations in the article are presented. Two entities are non-CAEs (see the shaded entities): Dopamine is not a specific target in the article, while transdihydrolisuride is an entity on which the findings may not be conclusive enough (see the underlined part).

As CAEs are the entities on which conclusions of an article are made, identification of CAEs is essential for the analysis of highly related conclusive findings in biomedical literature. Biomedical scientists are often concerned with conclusive findings on specific entities. For example, CTD, GHR (Genetic Home Reference), and OMIM (Online Mendelian Inheritance in Human) recruit many experts to frequently update their entity association databases by carefully searching for those articles whose main findings support the associations [2–4].

However, among the candidate entities in the title and the abstract of an article, identification of CAEs is challenging. For the article in Table 1, it is difficult to identify the *specific* targets and then estimate how *conclusive* the findings on the targets are (recall that Dopamine and transdihydrolisuride are not specific entities on which the reported findings are conclusive enough). For another example, consider the article in Table 2. This article mentions eight entities, and with this article, CTD curates two associations: the disease Parkinson’s disease has a ‘marker’ association with two chemicals MPTP and Trichloroethylene. The disease and the two chemicals are thus CAEs, and the other five entities are non-CAEs. These CAEs are discussed in different ways, and both CAEs and non-CAEs may appear at any parts of the article, including the title of the article. For example, Parkinsonism appears at the title of the article, but it is not a CAE (based on the curation done by CTD scientists). Parkinsonism refers to a group of neurological disorders that cause movement problems, but this article focuses on Parkinson’s disease specifically, because it investigates a neurodegeneration issue concerning Parkinson’s disease, which is a neurodegenerative brain disorder that causes the loss of motor control.

**Table 2:**
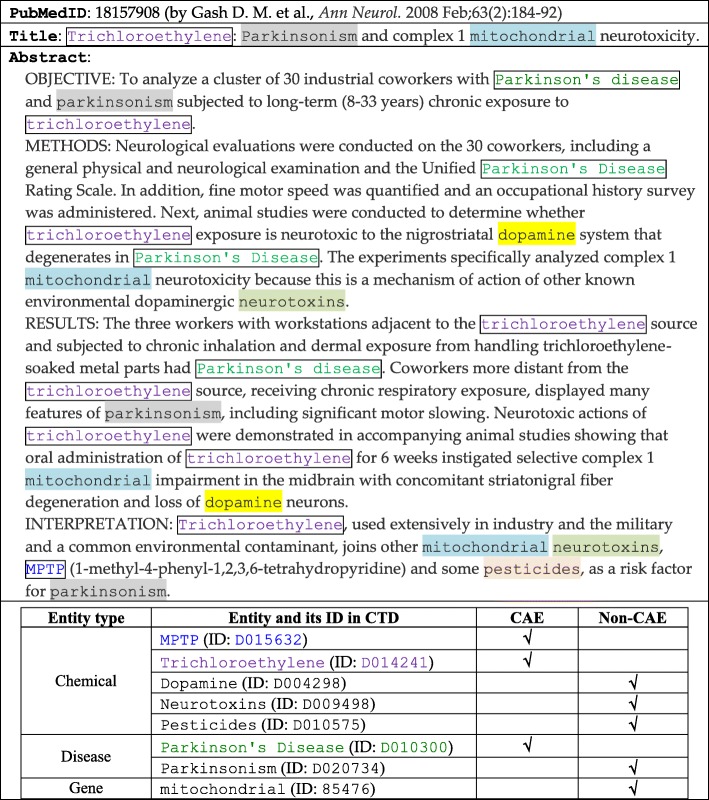
Another article curated by CTD. Three entities are identified as CAEs (see the boxed entities). More entities are not identified as CAEs (see the shaded entities), even though some of them appear at several places in the article, including the title.

One possible way to tackle the challenges of identifying CAEs is to build complete domain-specific knowledge, as well as intelligent and scalable discourse understanding techniques that can determine whether an entity is a *specific target* on which the reported findings are *conclusive enough*. However, it is both difficult and costly to build such domain-specific knowledge and intelligent techniques, and no previous studies built them to identify CAEs in biomedical articles.

## Problem definition and contribution

In this paper, we investigate the development of those techniques that, given candidate entities in the title and the abstract of a biomedical article *a*, identify CAEs in *a* for exploratory analysis. More specifically, we investigate how five types of statistical indicators can contribute to prioritizing the candidate entities so that CAEs can be ranked on the top, without relying on any domain knowledge and discourse analysis. These indicators include:

1. *Frequency-based* indicator: The indicator is concerned with the frequencies of candidate entities in article *a*. It is motivated by a hypothesis that CAEs in an article tend to appear frequently in the article. For example, in the examples discussed above, some CAEs have higher frequencies in the articles (e.g., 2-bromolisuride in Table 1 and trichloroethylene in Table 2).
2. *Rareness-based* indicator: The indicator is concerned with how rarely the candidate entities (in article *a*) appear in a collection of articles. An entity that appears in few articles is said to appear rarely. This indicator is motivated by a hypothesis that specific (general) entities tend to be rare (frequent) entity in articles. As noted above, specific entities in an article are likely to be CAEs in the article, making this indicator potentially helpful for CAE identification.
3. *Co-occurrence-based* indicator: The indicator is concerned with how often a candidate entity co-occurs with other entities in an article. It is motivated by a hypothesis that an entity that co-occurs with many other entities in an article may be related to these entities, and hence is likely to be a CAE in the article.
4. *Concentration-based* indicator: The indicator is concerned with how candidate entities (in article *a*) concentrate in a collection of articles. An entity that appears frequently in individual articles has a high concentration in these articles. This indicator is motivated by a hypothesis that an entity with a high concentration in articles may be a target of these articles, and hence it is likely to be a CAE of another article as well.
5. *Locality-based* indicator: The indicator is concerned with the positions of candidate entities in article *a*. It is motivated by a hypothesis that CAEs of an article may tend to be mentioned at certain parts that may be related to the goals and conclusions of the article. Such parts may include the title (e.g., prolactin in Table 1 and trichloroethylene in Table 2), the beginning part (e.g., 2-Br-LIS in Table 1 and Parkinson’s disease in Table 2), and the ending part (e.g., 2-Br-LIS in Table 1 and MPTP in Table 2) of the article.

Obviously, these indicators cannot always succeed in distinguishing CAEs from non-CAEs, because CAEs in an article may be discussed in different ways in different parts of the article. We thus have two research questions:

- (**Q1**) How does each indicator perform in identifying CAEs?
- (**Q2**) Can these indicators be fused to improve CAE identification?

We investigate these questions by those articles that biomedical experts believe to be targeted at specific associations among genes, diseases, and chemicals. Investigation of these questions can provide fundamental guidelines for the development of systems to index biomedical articles to support validation of highly related conclusive findings in biomedical literature.

## Related work

Our goal in this paper is to investigate how the five types of statistical indicators can be used to prioritize entities in titles and abstracts of articles so that CAEs, which are specific entities involved in the entity associations concluded in the articles, can be ranked on the top for exploratory analysis. To our knowledge, no previous studies focused on the same goal, and hence we discuss several types of related studies to clarify the contributions of the paper.

### Extraction of biomedical entity associations

CAEs are those entities that are involved in specific associations concluded in an article, and hence CAE identification is related to the task of extracting associations from the article. However, an association that happens to be mentioned in an article is *not* necessarily the conclusive finding of the article, due to two reasons: (1) the association may have been published, and it is mentioned in the article simply because it is related to the background of the article (rather than the main finding concluded in the article), and (2) the associated entities may not be the *specific* targets on which the reported findings are *conclusive enough* (e.g., the non-CAEs in the last sentence of the article shown in Table 2). Therefore, entities in an association extracted from an article are not necessarily CAEs of the article. We aim at prioritizing candidate entities so that CAEs in the articles can be ranked on the top.

Moreover, from a technical viewpoint, the statistical indicators investigated in this paper may provide different kinds of information to improve association extraction techniques, which often extracted associations by predefining a set of rules (e.g., [5–9]) and lexical-syntactic patterns (e.g., [7, 8, 10, 11]). As performance of association extraction was limited, various approaches were developed, such as integrating the rules and the patterns (e.g., [7–9, 12, 13]) and designing domain-specific rules and patterns (e.g., for protein-protein interaction [14], protein phosphorylation [15], and drug-drug interactions [16]). These previous techniques strived to design and tune the rule/pattern sets to consider the lexical, syntactic, semantic, anaphoric, and discourse aspects of understanding those sentences that might indicate associations. Instead of striving to understand these sentences, the indicators investigated in this paper rank CAEs based on statistical analysis on how candidate entities *individually* appear in the *whole* set of articles. Those entities that are ranked on the top in an article are likely to be entities of the associations reported in the article. These indicators may thus provide different types of information to further improve association extraction, without relying on a complete and scalable set of rules and patterns.

### Indexing of biomedical articles

CAEs are different from those MeSH (Medical Subject Heading) terms employed by PubMed to index articles. For example, for the article in Table 1, PubMed employs over ten MeSH terms as indexes, however many of them are not in the above set of CAEs (e.g., Animals and Ergolines) and some of the above CAEs are not employed as indexes (e.g., Hyperprolactinaemia and 2-bromolisuride). Index terms for an article are not necessarily those CAEs that biomedical experts employ to curate specific associations concluded in the article. Identification of CAEs in an article is thus different from indexing (labeling or classification) of the article with MeSH terms, which was a goal of many previous studies (e.g., techniques reported in the BioASQ workshop [17] and the Medical Text Indexer tool [18]).

### Ranking of entities

We model CAE identification as an *entity ranking* task, which aims at prioritizing candidate entities so that CAEs in an article can be ranked on the top. Many previous studies focused on entity ranking as well. However they have various goals different from ours in the paper.

Entity ranking was ever defined as a task to find a ranked list of entities that are of a specified type and have a certain relationship with a given entity [19, 20]. It was thus concerned with how a system ranked entities in response to a *query*, which consisted of three elements: an input entity, the type of the target entity, and a description of the relation. For example, to find “manufacturers of vehicles used by UPS”, the input entity may be “UPS”, the type of the target entity may be “manufacturer”, and the relation description may be “manufacturers of vehicles used by UPS” [20]. Many techniques were developed (e.g., [21]), and several variants of the problem scenarios were investigated, such as consdiering a chronologically ordered list of relevant documents [22] and providing support sentences for the entities retrieved [23]. When compared with these previous studies, we have a different goal: finding CAEs in a given article (rather than for a query). To identify the CAEs, no query is entered as input.

Another scenario of entity ranking was concerned with the ranking of entities in a given set *D* of documents, based on several factors such as the probability of the topics discussed in *D* as well as the correlation between the topics and the entities [24]. Therefore, its goal was to identify “popular” topic entities in *D*, while we have a different goal: finding those entities on which conclusive findings are reported (rather than popular topic entities) in an article (rather than a document collection).

Many previous studies aimed at ranking (extracting) entities (keywords) in an article as well, however their goals were different from ours as well. In the biomedical domain, the MetaMap indexing tool (MMI) was a component of Medical Text Indexer to index (label) articles with MeSH terms [18]. MMI only worked on MeSH terms in an article [25]. It employed the depth of each term in the MeSH tree as a critical factor to rank MeSH terms [25]. Therefore, effective techniques need to be developed to deal with those entities not in MeSH but in other ontologies (e.g., OMIM and the Entrez-Gene database, which are considered by curators of CTD [26]). We investigate potential contributions of five types of statistical indicators to identifying CAEs from various ontologies.

Another interesting feature of our goal is to identify CAEs in *titles* and *abstracts* of articles, which are more commonly available than *full texts* of the articles. Many previous studies worked on full texts of biomedical articles to identify important entities or keywords [27, 28]. For example, BioCreative defined entity ranking as a task of identifying important genes in a full-text article [27]. Important genes were those genes whose experimental settings contributed to main assertions of the article, and hence were essential for biomedical information curation [27]. Participants of BioCreative employed various strategies to rank genes, however many of the strategies cannot work well when only titles and abstracts are available (e.g., preferring those genes in the abstract, figure legends, table captions, or certain sections of the article [27]). As titles and abstracts are more commonly available than full texts, the techniques developed in the paper can be applicable to more articles. In the title and the abstract of an article, several entities may be related to experimental assertions of the article, but they are not necessarily CAEs, based on the curation done by CTD experts. Only *specific* entities on which *conclusive* findings are reported were selected as CAEs (recall that Lisuride was a CAE but Dopamine was not in Table 1; Parkinson’s disease was a CAE but Parkinsonism was not in Table 2).

We are thus concerned with the potential contributions of the five types of statistical indicators to ranking entities in titles and abstracts of articles. Some types of the indicators were considered by previous keyword rankers (extractors) as well. For example, a *frequency-based* indicator was employed to select keywords [25]. Integration of *frequency-based* and *rareness-based* indicators was one of the best techniques to extract keywords in articles [29, 30]. A *locality-based* indicator was employed by preferring those terms appearing in the title of a biomedical article [25]. A *co-occurrence-based* indicator was employed by keyword extractors in the biomedical domain [28], as well as other domains such as news [30, 31], computer science [32], and artificial intelligence [29].

When compared with these keyword rankers, we investigate how more types of indicators (and their fusion) perform in identifying those CAEs that are involved in the entity associations concluded in biomedical articles. Interestingly, we find that the indicators do not necessarily perform well in identifying the CAEs, and learning-based fusion of the indicators can further improve performance (ref. Results).

### Retrieval of articles for specific entities

Retrieval of relevant articles for a query term (entity) is often based on the estimation of the *relatedness* between the term and each article. A CAE identifier requires such a relatedness estimation component as well. However, when compared with article retrievers, instead of retrieving articles for a query entity, a CAE identifier conversely finds entities that are related to the conclusive findings of a given article. Therefore, although article retrievers do not aim at CAE identification, some of their term-article relatedness components may have potential contributions to CAE identification.

The *frequency-based* and the *rareness-based* indicators were routinely considered by biomedical article retrievers. Among the previous article retrievers that considered the two indicators, BM25 [33] was one of the best techniques in finding biomedical articles [34]. A *concentration-based* indicator was considered by an article retriever ES, which was tested in [35] and found to be one of the best biomedical articles retrievers [36]. *Locality-based* indicators were employed by many article retrievers, which preferred those articles in which the entities of interest appeared at certain parts of the articles, including the titles, the first sentences, and the last sentences of the articles [26, 37, 38]. Similar locality information was employed to retrieve articles about specific gene-disease associations [39] and estimate inter-article similarity [40]. The locality-based information was also used to extract text passages (e.g., sentences) about gene functions [41] and evidence-based medicine [42].

Note that the previous article retrievers also employed several indicators that are helpful for article retrieval but *not* CAE identification. We thus do not investigate them in this paper. For example, PubMed considered the query length as an indicator to improve article retrieval [38]. This indicator is *query-specific* without providing helpful information to CAE identification in which no input query is assumed. Similarly, we do not investigate *article-specific* indicators, such as the article length, as well as the field length (e.g., the lengths of the title and the abstract), publication type, and publication year, which were considered by PubMed [38]. They are not helpful for CAE identification, which aims at finding CAEs in a given article, rather than ranking multiple articles with different article-specific characteristics.

It is thus interesting to identify those indicators that have potential contributions to CAE identification, and investigate how they really perform in CAE identification. We identify the five types of indicators based on the observation of how CAEs may appear in biomedical articles. These indicators are investigated both *individually* and *collectively*, and case studies are conducted to further investigate their practical contributions to curation of biomedical databases.

## Methods

The steps to conduct the research include (1) selection of the potential indicators for CAE identification, (2) fusion of the indicators, and (3) performance evaluation.

### Potential indicators

Table 3 defines the five types of indicators investigated in the paper. The first indicator is *TF* (term frequency), which is a frequency-based indicator. It counts the number of times an entity appears in an article. As CAEs in an article may appear frequently in the article, one may expect that an entity with a high TF is likely to be a CAE of the article. The second indicator is *IDF* (inverse document frequency), which is a rareness-based indicator. An entity that appears in fewer articles will have a larger *IDF*, which may also indicate that the entity is more specific. As CAEs in an article *a* tend to be specific ones, we expect that an entity with a higher *IDF* is likely to be a CAE in *a*.

**Table 3:** Definitions of individual indicators

Type	Indicator	Definition
(1) *Frequency-based*	*TF*	*TF*(*e*, *a*) = *Number of times e appears in a*
(2) *Rareness-based*	*IDF*	$IDF(e)={Log}_2\frac{\mid A\mid +{1}^{\left[\mathrm{i}\right]}}{DF(e)+{1}^{\left[\mathrm{i}\mathrm{i}\right]}}$
(3) *Co-occurrence-based*	*CoOcc*	$CoOcc\left(e,a\right)=\sum \limits_{x\in a,x\ne e}\frac{{\left|{S}_{e\cap x}(a)\right|}^{\left[\mathrm{iii}\right]}}{{\left|{S}_e(a)\right|}^{\left[\mathrm{iv}\right]}}$
(4) *Concentration-based*	*AvgTF*	$AvgTF(e)=\frac{c{\left(e,C\right)}^{\left[\mathrm{v}\right]}}{DF(e)}$
(5) *Locality-based*	*TITLE*	$$ TITLE\left(e,a\right)=\left\{\begin{array}{c}1,\mathrm{if}\ \mathrm{e}\ \mathrm{a}\mathrm{ppears}\ \mathrm{in}\ \mathrm{title}\ \mathrm{of}\ \mathrm{a};\\ {}0,\mathrm{otherwise}.\end{array}\right. $$
	*AbstractX*	$$ AbstractX\left(e,a\right)=\left\{\begin{array}{c}1,\mathrm{if}\ \mathrm{e}\ \mathrm{a}\mathrm{ppears}\ \mathrm{in}\ \mathrm{the}\ \mathrm{first}\ \mathrm{X}\ \mathrm{or}\ \mathrm{Last}\ \mathrm{X}\\ {}\ \mathrm{sentences}\ \mathrm{in}\ \mathrm{abstract}\ \mathrm{of}\ \mathrm{a};\\ {}0,\mathrm{otherwise}.\end{array}\right. $$

^[i]^ |*A*| = Number of articles in the collection of articles *C*;

^[ii]^*DF*(*e*) = Number of articles (in *C*) mentioning *e*;

^[iii]^*S*_*e*∩*x*_(*a*) = Set of sentences (in *a*) that *e* and *x* co-occur;

^[iv]^*S*_*e*_(*a*) = Set of sentences (in *a*) mentioning *e*;

^[v]^*c*(*e*,*C*) = Number of times *e* appears in articles in *C*

The third indicator is *CoOcc*, which is a co-occurrence-based indicator. Following [28], it is defined in Eq. 1. For an entity *e* in an article *a*, *CoOcc* is the sum of the probabilities of *e* co-occurs with other entities in sentences in *a*. One may expect that an entity with a larger *CoOcc* in an article *a* may be related to more entities in *a*, and hence is likely to be a CAE in *a*.

The fourth indicator is *AvgTF*, which is a concentration-based indicator. For an entity *e*, *AvgTF* is the micro average frequency of *e* appearing in a collection of articles. An entity with a larger *AvgTF* in a collection of articles may be a target of these articles, and hence it is likely to be a CAE of articles as well.

The fifth and the sixth indicators are *TITLE* and *AbstractX*, which are locality-based indicators. For an entity *e* in an article *a*, *TITLE* is concerned with whether *e* appears in the title of *a*, while *AbstractX* is concerned with whether *e* appears in the first X or last X sentences in the abstract of *a*. As the title, the first sentences, and the last sentences of an article are often treated as critical parts for retrieval of biomedical articles [26, 37, 38], one may expect that an entity with larger *TITLE* and *AbstractX* is likely to be a CAE of *a*.

### Fusion of the indicators

Proper fusion of the above indicators may improve CAE identification. We thus investigate two kinds of fusion strategies: *learning-based strategies* and *typical strategies*. For the learning-based strategies, we employ RankingSVM [43], which is one of the best techniques routinely used to integrate multiple indicators by SVM (Support Vector Machine) to achieve better ranking (e.g., [36, 44]). We employ SVM^*rank*^ [45] to implement RankingSVM. All the indicators are integrated by RankingSVM. Different combinations of the indicators are also tested to identify the best ways to fuse the indicators.

For the typical fusion strategies, Table 4 summarizes several indicators that are defined based on state-of-the-art keyword extractors and article retrievers. The first type of typical strategies fused the frequency-based (*TF*) and rareness-based (*IDF*) indicators. TFIDF and BM25*e* are two indicators of this type. TFIDF is the product of *TF* and *IDF*. It was found to be one of the best techniques to extract keywords in articles [29, 30]. BM25*e* is defined based on BM25, which was found to be one of the best techniques to retrieve biomedical articles [34]. It employs Eq. 1 to estimate the similarity between and entity *e* and an article *a*, where *k*_1_ and *b* are two parameters, *|a*| is the number of terms in article *a* (i.e., length of *a*), and *avgal* is the average length of a collection of articles.

1 $$ BM25e\left(e,a\right)=\frac{TF\left(e,a\right)\left({k}_1+1\right)}{TF\left(e,a\right)+{k}_1\left(1-b+b\frac{\left|a\right|}{avgal}\right)} IDF(e) $$

**Table 4:**
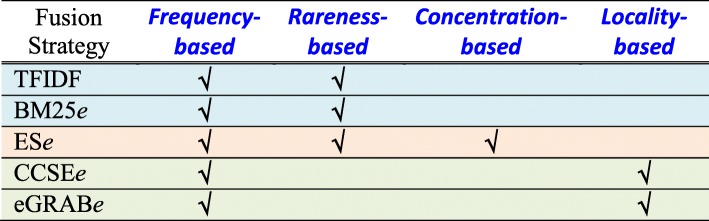
Typical strategies to fuse the indicators

The second type of typical strategies fused frequency-based, rareness-based, and concentration-based indicators. ES*e* is an indicator of this type. It is defined based on ES, which was one of the best techniques to retrieve biomedical articles as well [36]. ES*e* employs Eq. 2 to estimate the similarity between and entity *e* and an article *a*, where, where *DF*(*e*) is the number of articles containing *e* (i.e., *document frequency* of *e*); *C* is a collection of articles; *N* is the total number of articles in *C*; *c*(*e*,*C*) is the number of times *e* appears in *C*. Therefore, ES*e* implements a concentration-based indicator by the ratio of *c*(*e*,*C*) to *DF*(*e*), which measures how *e* concentrates in articles by computing the micro average TF of *e* in the articles.

2 $$ ESe\left(e,a\right)=\frac{TF\left(e,a\right)}{TF\left(e,a\right)+0.45\cdot \sqrt{\frac{\left|a\right|}{avgdl}}}\cdot \sqrt{{\left(\frac{c\left(e,C\right)}{DF(e)}\right)}^3\cdot \frac{N}{DF(e)}} $$

The third type of typical strategies fused frequency-based and locality-based indicators. CCSE*e* and eGRAB*e* are two indicators of this type. CCSE*e* is defined based on a locality-based biomedical article retriever CCSE (core content similarity estimation [40]). The CCSE*e* score of an entity *e* in an article *a* is the sum of three factors concerning how *e* is related to the goal, background, and conclusion of *a*. The factors are defined as linear weights that are derived based on the positions of *e* in *a* (for detailed definitions for the linear weights, the reader is referred to [40]). For example, *e* is related to the goal of *a* if it occurs in the title of *a*; *e* is related to the background of *a* if it occurs in the beginning part of the abstract of *a*; and *e* is related to the conclusion of *a* if it occurs in the ending part of the abstract of *a*. Similarly, eGRAB*e* considers locality information as well. It is defined based on a gene article retriever eGRAB (extractor of gene-relevant abstracts [37]). The eGRAB*e* score of an entity *e* in an article *a* is increased by 1 if (1) *e* appears in *a* at least three times; (2) *e* appears in the title of *a*; or (3) *e* appears in first X or last X sentences in the abstract of *a*. We set X to 1 ~ 3, and hence have three respective versions: eGRAB*e*-1, eGRAB*e*-2, eGRAB*e*-3. Note that, in addition to the locality-based information of an entity, both CCSE*e* and eGRAB*e* have incorporated frequency-based information as well, because an entity with multiple occurrences in different parts of an article will get amplified scores.

### Performance evaluation

#### The data

Experimental data is collected from CTD (available at http://ctdbase.org/), which recruits biomedical experts to maintain a database of biomedical articles with main research focuses on associations between chemicals, genes, and diseases [1, 26]. CTD recruited and trained a number of biomedical experts to curate the associations with a controlled vocabulary. An experiment showed that the experts achieved a high degree of agreement in selecting articles to curate (77% agreement among all curators, and 85% average agreement between every two curators), with good accuracy in curating associations in the articles (average precision and recall were 0.91 and 0.71 respectively) [26]. Associations curated by CTD experts are also reviewed for quality control before they are released [26].

We thus evaluate how CAEs curated by the experts are identified by systems. We randomly sample 300 entities from three kinds of association files in CTD: <chemical, gene>, <chemical disease>, and < gene, disease>. For each entity *e* all associations involving *e* are collected. These associations can serve as the basis to comprehensively collect test articles. For each of the associations, we collect all articles that CTD experts selected to curate the association. For each article *a*, we collect *all* associations that CTD experts curated with *a*. Entities involved in these associations can thus be the CAEs in *a* (i.e., the gold standard for *a*). We totally have 60,507 articles with their CAEs appearing in their titles or abstracts (see Additional file 1). These articles amount to about 50% of all articles in CTD.

As we are evaluating how systems perform in identifying CAEs among a given set of candidate entities in an article, candidate entities in each article should be identified. For our evaluation purpose, the candidate entities need to be identified based on the vocabulary of CTD, because CTD experts have employed this vocabulary to curate CAEs in the articles. Other potential entities not in the vocabulary are beyond the scope of consideration, because whether they are CAEs in the articles is *not* verified by domain experts.

More specifically, this vocabulary comprehensively includes about 2.5 million (2,535,754) terms for the names, symbols, and synonyms of entities of three types: genes, diseases, and chemicals. They are selected and modified from multiple sources, such as MeSH (for chemicals and diseases), the Entrez-Gene database (for genes, developed by National Center for Biotechnology Information), and OMIM (for diseases) [26]. The vocabulary is thus “customized” for the curation purpose of CTD (e.g., entities for species-specific entities are added, while some entities not considered by CTD are removed [46–48]). Candidate entities in each article are mapped to their IDs by a dictionary-based normalization approach, which was employed by many previous studies as well (e.g., [6, 49, 50]). To further fit the approach to our evaluation purpose, given an article *a*, all terms that are CAEs of *a* are first mapped to their corresponding entity IDs, as the existence of the entities in *a* has been confirmed by CTD experts. Other terms are then identified by checking whether official symbols or names of entities in the vocabulary appear in *a*; and if no, synonyms of entities are checked. Moreover, authors of articles often employ their own abbreviations (or symbols) to represent an entity. For example, the article in Table 1 contains several “author-defined” abbreviations expressed in parentheses, such as DA (for dopamine), LIS (for lisuride), and TDHL (for transdihydrolisuride). We thus map these abbreviations to their corresponding entity IDs as well.

As noted above (ref. Fusion of the indicators), we also investigate several learning-based strategies to fuse individual indicators, and hence we require training data to train the fusion systems. We thus evenly split the 60,507 articles into five parts on which 5-fold cross validation is conducted. In each experiment fold, a part of the data is used for testing while the other four parts are used for training, and the cross-validation process is repeated five times, with each of the five parts being used exactly once as testing data.

#### Evaluation criteria

As the systems aim at prioritizing candidate entities in an article so that CAEs of the article can be ranked on the top, we employ three evaluation criteria to measure how CAEs are ranked high. The first criterion is *mean average precision* (MAP), which is defined in Eq. 3, where |A| is the number of test articles in the experiment (i.e., |A| = 60,507), *k*_*i*_ is number of entities that are believed (by CTD experts) to be CAEs of the *i*^th^ article, and *Seen*_*i*_(*j*) is the number of entities whose ranks are higher than or equal to that of the *j*^th^ CAE for the *i*^th^ article. Therefore, *AP*(*i*) is actually the average precision (AP) for the *i*^th^ article. It is the average of the precision when each CAE is seen in the ranked list. Given an article, if a system can rank higher those CAEs in the article, AP for the article will be higher. MAP is simply the average of the AP values for all test articles.

3 $$ MAP=\frac{\sum \limits_{i=1}^{\mid \mathrm{A}\mid } AP(i)}{\mid \mathrm{A}\mid },\kern1em AP(i)=\frac{\sum \limits_{j=1}^{k_i}\frac{j}{Seen_i(j)}}{k_i} $$

The second criterion is *average precision at top-X* (Average P@X, see Eq. 4), which is the average of the P@X values for all test articles. P@X is the precision when top-X entities are shown to the readers (see Eq. 5). Therefore, when X is set to a small value, P@X measures how a system ranks CAEs very high. In the experiments, we set X to 1, 3, and 5.

4 $$ \mathrm{A}\mathrm{verage}\kern0.5em \mathrm{P}@\mathrm{X}=\kern0.5em \frac{\sum \limits_{i=1}^{\mid \mathrm{A}\mid}\mathrm{P}@\mathrm{X}(i)}{\mid \mathrm{A}\mid } $$

5 $$ \mathrm{P}@\mathrm{X}(i)=\kern0.5em \frac{\mathrm{Number}\kern0.17em \mathrm{of}\ \mathrm{top}\hbox{-} \mathrm{X}\;\mathrm{entities}\kern0.5em \mathrm{that}\;\mathrm{are}\ \mathrm{CAEs}\kern0.17em \mathrm{in}\kern0.5em \mathrm{the}\kern0.5em {i}^{th}\;\mathrm{article}}{\mathrm{X}} $$

The third evaluation criterion is *%P@X > 0*, which is the percentage of the test articles that have at least one CAE ranked at top-X positions (X = 1, 2 and 3). It can be a good measure to indicate whether a system can successfully identify CAEs for a large portion of the test articles. This measure is of practical significance, because a CAE identification system can provide practical support to biomedical researchers only if it can successfully identify CAEs for most articles.

## Results

We separately present the experimental results, which aim at answering the two research questions (Q1 and Q2) respectively. Case studies are also conducted to show how the identified CAEs can be visualized to support exploratory analysis for curating biomedical databases.

### Q1: How does each indicator perform in identifying CAEs?

**Fig. 1:**
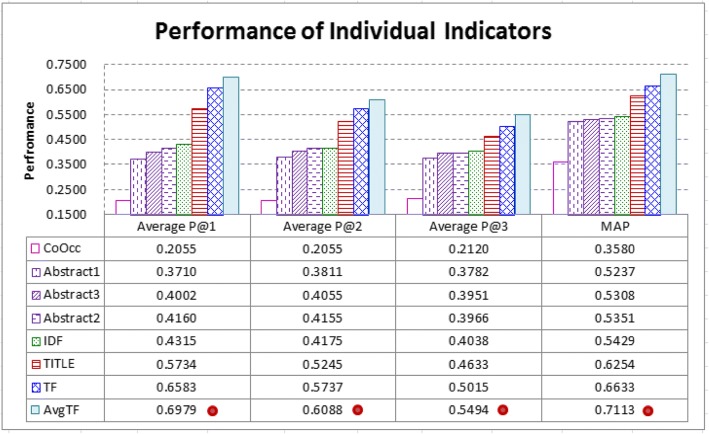
Performance of individual indicators: The *concentration*-based indicator (i.e., *AvgTF*) performs significantly better than all the other indicators (‘•’ denotes that the indicator performs significantly better than others)

Figure 1 shows the performance of each individual indicator in CAE identification. To verify whether the performance differences between two indicators are statistically significant, we conduct paired t-test with 99% as the confidence level. The results show that the *concentration*-based indicator (i.e., *AvgTF*) performs significantly better than all the other indicators.

We further analyze each indicator by investigating how CAEs and non-CAEs distribute with the information provided by each indicator. For an indicator *c*, Eq. 6 is used to compute the probability of CAEs whose values (estimated by *c*) fall in a specific interval. Similarly, Eq. 7 is defined for the probability of non-CAEs. Therefore, given an indicator *c*, these probabilities aim at measuring the “prevalence rate” of CAEs and non-CAEs in each interval. Moreover, we are also concerned with the *probability gain* of finding CAEs (*ProbGain*) in each interval (see Eq. 8). It is the difference between the probability of finding CAEs in an interval and the overall probability of finding CAEs. Therefore, a positive (negative) *ProbGain* in an interval indicates that it is generally more (less) likely to find CAEs in the interval.

6 $$ {\mathrm{P}}_i\left(\mathrm{CAE}\right)=\kern0.5em \frac{\mathrm{Number}\kern0.5em \mathrm{of}\kern0.5em \mathrm{CAEs}\kern0.5em \mathrm{that}\kern0.5em \mathrm{fall}\kern0.5em \mathrm{in}\kern0.5em \mathrm{in}\mathrm{terval}\kern0.5em i}{\mathrm{Total}\kern0.5em \mathrm{number}\kern0.5em \mathrm{of}\kern0.5em \mathrm{CAEs}\kern0.5em \mathrm{in}\kern0.5em \mathrm{all}\kern0.5em \mathrm{articles}} $$

7 $$ {\mathrm{P}}_i\left(\mathrm{NonCAE}\right)=\kern0.5em \frac{\mathrm{Number}\kern0.5em \mathrm{of}\kern0.5em \mathrm{non}\hbox{-} \mathrm{CAEs}\kern0.5em \mathrm{that}\kern0.5em \mathrm{fall}\kern0.5em \mathrm{in}\kern0.5em \mathrm{in}\mathrm{terval}\kern0.5em i}{\mathrm{Total}\kern0.5em \mathrm{number}\kern0.5em \mathrm{of}\kern0.5em \mathrm{non}\hbox{-} \mathrm{CAEs}\kern0.5em \mathrm{in}\kern0.5em \mathrm{all}\kern0.5em \mathrm{articles}} $$

8 $$ {ProbGain}_i=\kern0.5em \frac{\mathrm{Number}\kern0.5em \mathrm{of}\kern0.5em \mathrm{CAEs}\kern0.5em \mathrm{in}\kern0.5em \mathrm{in}\mathrm{terval}\kern0.5em i}{\mathrm{Number}\kern0.5em \mathrm{of}\kern0.5em \mathrm{entities}\kern0.5em \mathrm{in}\kern0.5em \mathrm{in}\mathrm{terval}\kern0.5em i}-\frac{\mathrm{Number}\kern0.5em \mathrm{of}\kern0.5em \mathrm{CAEs}\kern0.5em \mathrm{in}\kern0.5em \mathrm{all}\kern0.5em \mathrm{articles}}{\mathrm{Total}\kern0.5em \mathrm{number}\kern0.5em \mathrm{of}\kern0.5em \mathrm{entities}\kern0.5em \mathrm{in}\kern0.5em \mathrm{all}\kern0.5em \mathrm{articles}} $$

**Fig. 2:**
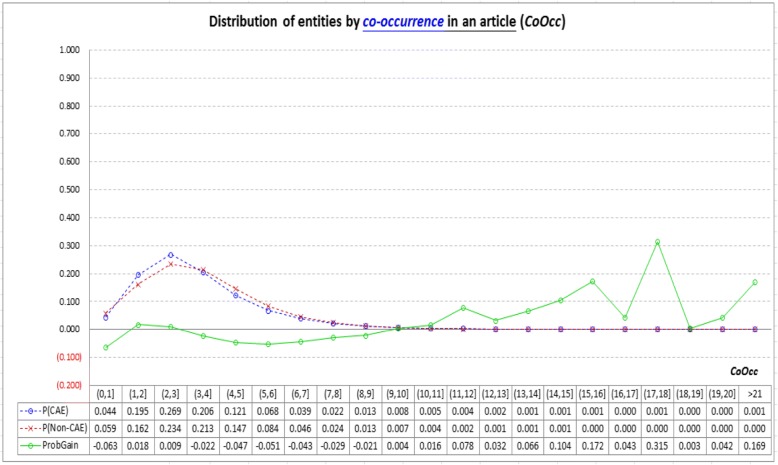
Analysis of the *co-occurrence*-based indicator (*CoOcc*): Both CAEs and non-CAEs mainly fall in the area where 0 < *CoOcc* ≤ 10 (see the two dashed lines), however in this area, probability gain of finding CAEs (*ProbGain*) oscillates around zero with small absolute values (see the solid line), and hence most CAEs and non-CAEs have similar *CoOcc* values. This is the reason why *CoOcc* is less capable of distinguishing CAEs from non-CAEs

Figure 2 shows how CAEs and non-CAEs distribute with the information provided by *CoOcc*. The two dashed lines respectively show the prevalence probabilities of CAEs and non-CAEs. They indicate that CAEs and non-CAEs mainly fall in the area where 0 < *CoOcc* ≤ 10. However in this area, *ProbGain* oscillates around zero with small absolute values. Therefore, most CAEs and non-CAEs have similar *CoOcc* values, making *CoOcc* less capable of distinguishing CAEs from non-CAEs. Given that co-occurrence-based information was found to be one of the best information to extract keywords [29, 30], the result show that it is *not* necessarily quite helpful for identifying CAEs in biomedical articles.

**Fig. 3:**
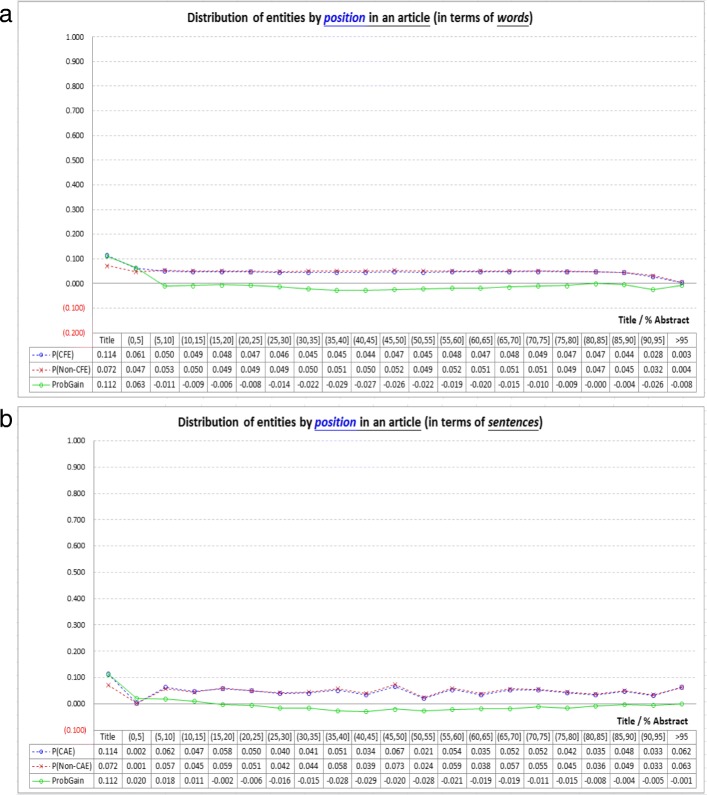
Analysis of the *locality*-based indicators (*AbstractX* and *TITLE*): Positions of entities can be measured in terms of words or sentences, as shown in (*a*) and (*b*) respectively. When compared with non-CAEs, CAEs are more likely to appear in *titles* of articles, as shown in the left-most parts of (*a*) and (*b*). On the other hand, when considering the *abstracts* of the articles, both CAEs and non-CAEs have somewhat uniform distributions at different positions, and moreover they have very similar distributions (see the two overlapping dashed lines). Therefore, *TITLE* works better than *AbstractX* in distinguishing CAEs from non-CAEs (see the solid line). However, *TITLE* has weaknesses as well, because most CAEs do not appear in the titles of articles (only 11.4% of CAEs appear in the titles)

Figure 3 shows how CAEs and non-CAEs distribute with the information provided by the *locality*-based indicators (i.e., *AbstractX* and *TITLE*). Positions of entities can be measured in terms of words or sentences. An abstract is divided into 20 parts, and in each part we compute the prevalence probabilities of CAEs and non-CAEs, as well as *ProbGain*. The results show that, when considering the *abstracts* of the articles, both CAEs and non-CAEs have somewhat uniform distributions at different positions, and they have very similar distributions (and hence *ProbGain* in the abstract parts oscillates around zero with small absolute values). Therefore, although the first sentences and the last sentences of abstracts were used to retrieve biomedical articles [26, 37, 40], they are not necessarily quite helpful for CAE identification. On the other hand, *TITLE* works better, as CAEs are more likely to appear in *titles* than non-CAEs. However, *TITLE* has weaknesses as well, because most CAEs do not appear in titles (as shown in the leftmost part of Fig. 3, only 11.4% of CAEs appear in titles).

**Fig. 4:**
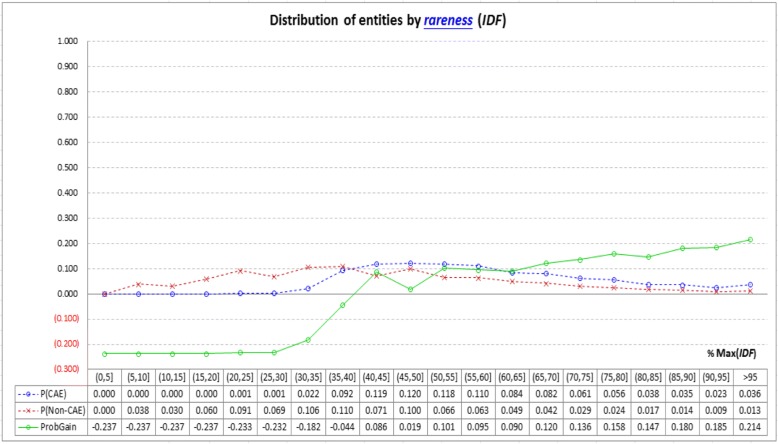
Analysis of the *rareness*-based indicator (*IDF*): *IDF* values of non-CAEs fall in the whole *IDF* spectrum, while nearly no CAEs have low *IDF* values (see the two dashed lines). *IDF* may thus be a useful indicator for CAE identification, especially for those entities with lower *IDF* values (i.e., the lower 30% of the *IDF* spectrum, see the solid line for probability gain of finding CAEs). However, many entities have *IDF* values fall in the middle parts of the spectrum (i.e., between 35 and 65%). For these entities, *IDF* may not work well (see the solid line for *ProbGain*)

Figure 4 shows how CAEs and non-CAEs distribute with the information provided by the *rareness*-based indicators (i.e., *IDF*). The *IDF* spectrum is divided into 20 parts. *IDF* values of non-CAEs fall in the whole spectrum, however nearly no CAEs have *IDF* values falling in the lower 30% part. *ProbGain* of *IDF* thus oscillates more dramatically than those of the co-occurrence-based and locality-based indicators noted above, making *IDF* more helpful for CAE identification, especially for those entities with lower *IDF* values. However, many entities have *IDF* values fall in the middle parts of the spectrum (i.e., between 35 and 65%). For these entities, *IDF* may not work well.

**Fig. 5:**
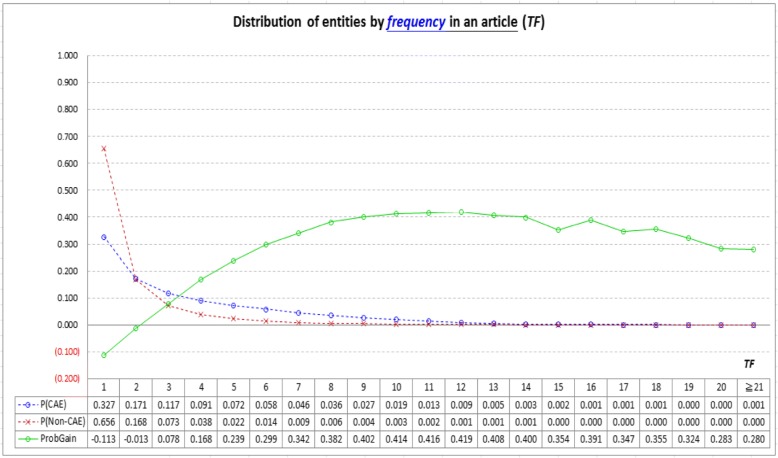
Analysis of the *frequency*-based indicator (*TF*): Most entities have *TF* less than 6 (see the two dashed lines). Most non-CAEs have a TF value equal to 1, and probability gain of finding CAEs (*ProbGain*) becomes large when *TF* ≥ 4 (see the solid line). *TF* thus performs well in identifying CAEs whose *TF* = 1 or *TF* ≥ 4. However, many entities have *TF* values falling between 2 and 3, and for these entities *TF* has difficulty in distinguishing CAEs from non-CAEs

Figure 5 shows how CAEs and non-CAEs distribute with the information provided by the *frequency*-based indicator (*TF*). Most entities have *TF* values less than 6. Most non-CAEs have TF = 1, and *ProbGain* of finding CAEs becomes large when *TF* ≥ 4 (see the solid line). *TF* thus performs well in identifying CAEs whose *TF* = 1 or *TF* ≥ 4. However, many entities have *TF* values falling between 2 and 3, and for these entities *TF* has difficulty in distinguishing them (absolute value of *ProbGain* is small for *TF* = 2 or 3). Therefore, although it is reasonable to retrieve articles for an entity by preferring those articles in which the entity appears at least three times [37], this strategy may not be suitable for identifying CAEs.

**Fig. 6:**
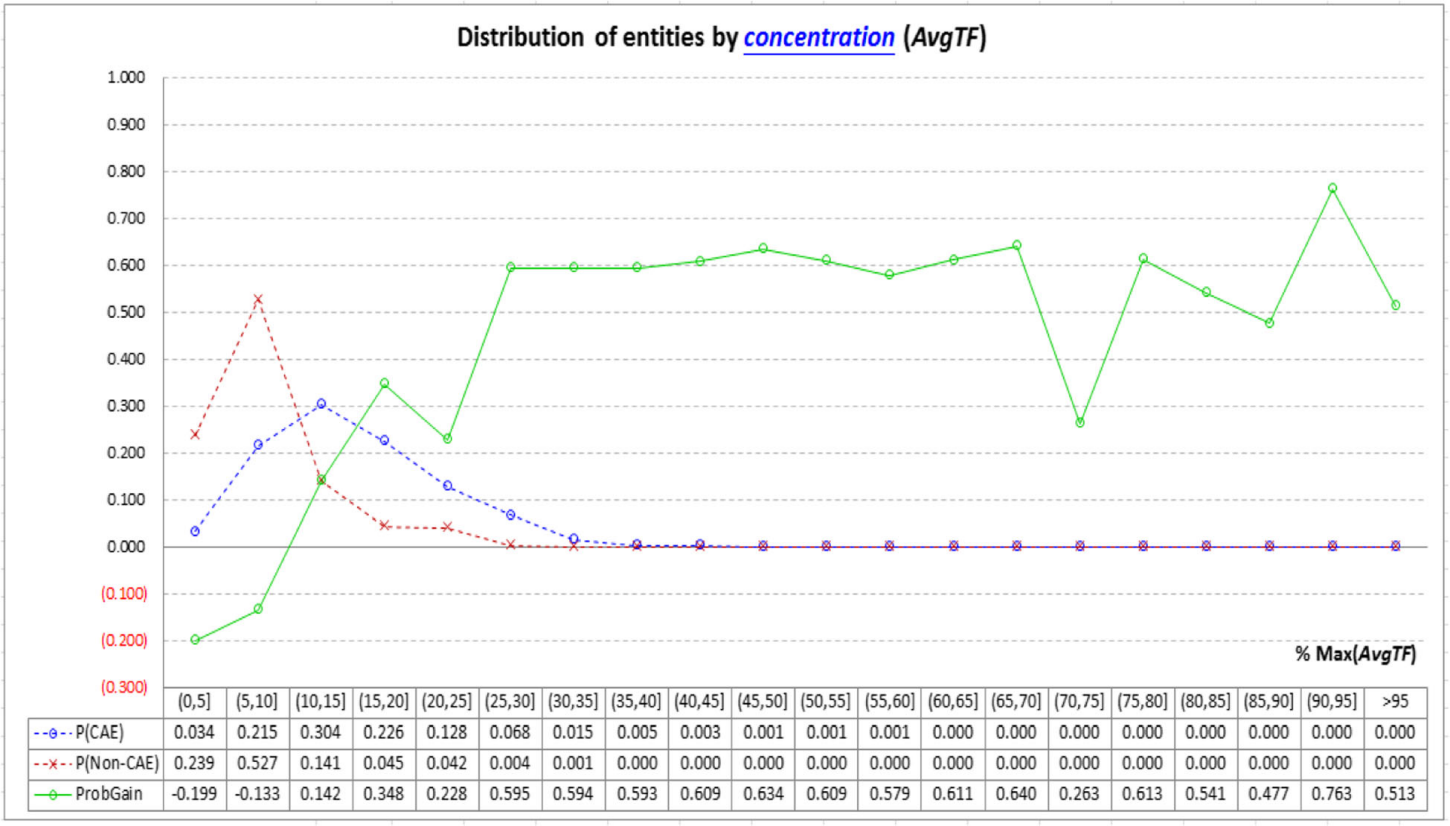
Analysis of the *concentration*-based indicator (*AvgTF*): Most CAEs have *AvgTF* values falling between 10 to 40% of the maximum, while most non-CAEs have *AvgTF* values falling below 10% of the maximum (see the two dashed lines). Therefore, probability gain of finding CAEs (*ProbGain*) oscillates more dramatically with larger absolute values (see the solid line), and hence *AvgTF* performs better than all the other indicators in CAE identification

Figure 6 shows how CAEs and non-CAEs distribute with the information provided by the *concentration*-based indicator (*AvgTF*). The *AvgTF* spectrum is divided into 20 parts, and most CAEs have *AvgTF* values falling between 10 to 40% of the maximum *AvgTF*, while most non-CAEs have *AvgTF* values falling below 10% of the maximum *AvgTF*. Therefore, when compared with other indicators, *AvgTF* has *ProbGain* that oscillates more dramatically, making it more capable of distinguishing CAEs from non-CAEs.

Table 5 summarizes the potential and the limitation of each indicator in CAE identification. In conclusion, these indicators have significantly different performance in CAE identification. *AvgTF* has significantly better performance than all other indicators. Concentration of an entity in a collection of articles is thus a good way to distinguish CAEs from non-CAEs. *CoOcc* and *AbstractX* are less capable of distinguishing CAEs from non-CAEs, although they have been used in many article retrievers and keyword extractors. Other indicators may have their own weaknesses as well, especially when identifying CAEs with different statistical characteristics.

**Table 5:** Summary of the performance of each indicator

Indicator	Potential in CAE identification	Limitation in CAE identification
*TF*	*TF* works well for those entities whose *TF* = 1 or *TF* ≥ 4, as non-CAEs tend to have *TF* = 1, and few of them have *TF* ≥ 4.	Many CAEs and non-CAEs have *TF* values falling between 2 and 3.
*IDF*	*IDF* values of non-CAEs fall in the *IDF* spectrum, while nearly no CAEs have *IDF* values falling in the lower 30% part, making *IDF* helpful to filter out non-CAEs with lower *IDF* values.	Many CAEs and non-CAEs have *IDF* values fall in the middle parts of the spectrum (i.e., between 35 and 65%).
*CoOcc*	None.	CAEs and non-CAEs tend to have similar *CoOcc* values.
*AvgTF*	CAEs tend to have *AvgTF* > 10% of the maximum *AvgTF*, while non-CAEs tend to have *AvgTF* ≤ 10% of the maximum.	None.
*TITLE*	When compared with non-CAEs, CAEs are more likely to appear in titles of articles.	Most CAEs do not appear in the titles of articles.
*AbstractX*	None.	CAEs and non-CAEs have somewhat uniform and similar distributions at different positions in the abstract.

### Q2: Can these indicators be fused to improve CAE identification?

**Fig. 7:**
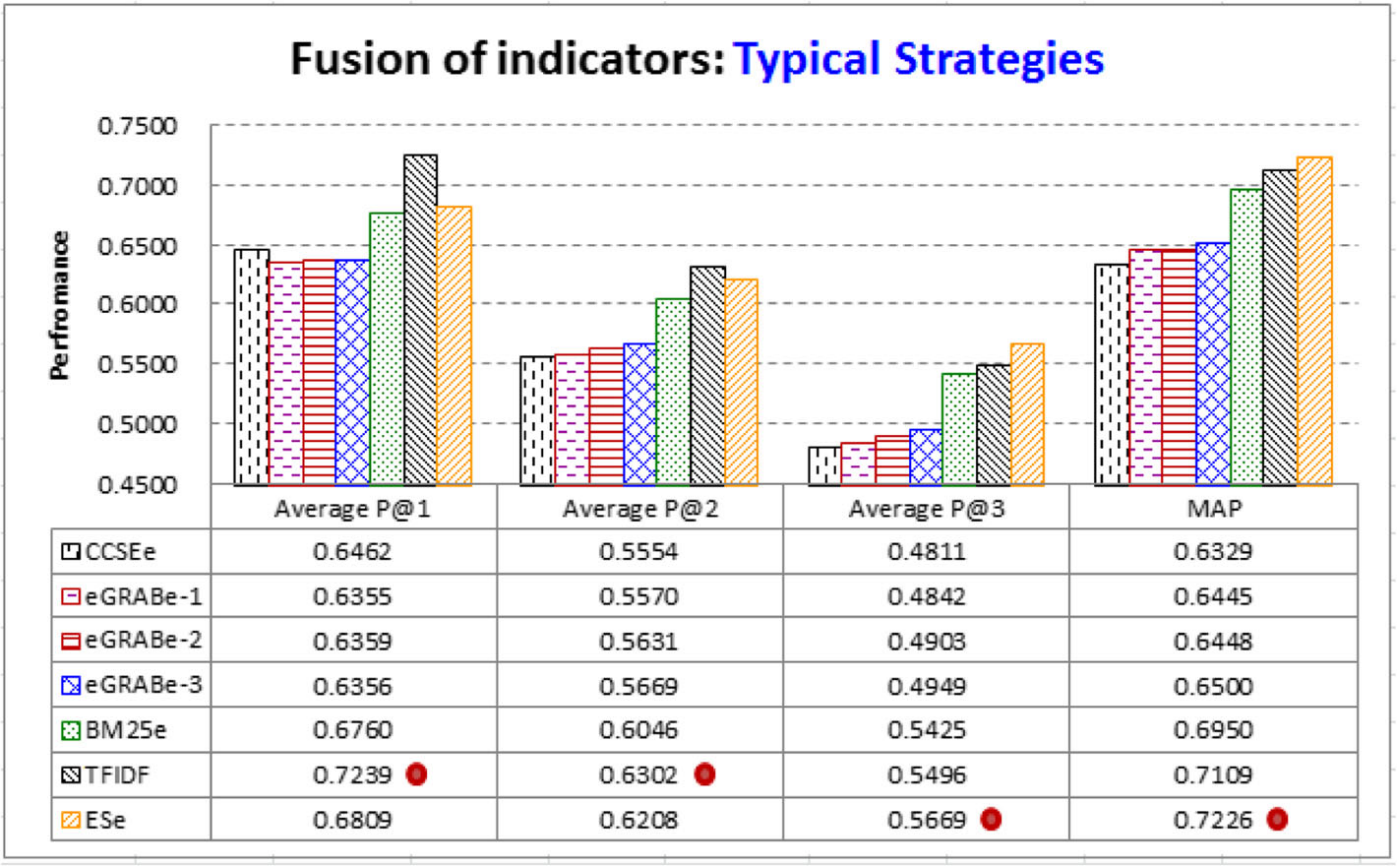
Fusion of indicators by typical strategies: All the typical strategies have considered the frequency-based indicator (*TF*). However, CCSE*e* and eGRAB*e*, which fuse *TF* and locality-based indicators, even deteriorates performance (*TF* has been able to achieve a higher MAP of 0.6633, ref. Fig. 1). BM25*e* and TFIDF, which fuse *TF* and the rareness-based indicator *IDF*, can further improve performance. TFIDF performs significantly better than others in Average P@1 and P@2 (‘•’ denotes that the indicator performs significantly better than others). ES*e* fuses *TF*, *IDF*, and the concentration-based indicator (*AvgTF*). It performs significantly better than others in Average P@3 and MAP

It is thus interesting to fuse the indicators to further improve performance. A poorer indicator may still contribute, especially if it can provide helpful information that is not provided by other indicators. Figure 7 shows the performance of the typical fusion strategies. As noted above (ref. Fusion of the indicators), all the fusion strategies consider *TF*, however they have significantly different performance.

CCSE*e* and eGRAB*e*, which considers both *TF* and locality-based information, perform even worse than *TF*. They have lower MAP than *TF* (CCSE*e*: 0.6329; eGRAB*e*-3: 0.6500; but *TF*: 0.6633, ref. Fig. 1). As noted above (ref. Fusion of the indicators), CCSE*e* and eGRAB*e* are respectively based on two article retrievers CCSE [40] and eGRAB [37]. They consider *TF* and *TITLE*, which are helpful indicators in certain cases (ref. Fig. 3, and ref. Fig. 5). However, *TF* has weaknesses as well because many CAEs and non-CAEs have *TF* values falling between 2 and 3 (as noted in the discussion for Fig. 5). CCSE*e* and eGRAB*e* cannot properly tackle this weakness, even though they also consider the positions of entities in the abstract, which are less helpful for CAE identification (ref. the poor performance of *AbstractX*, noted in the discussion for Fig. 3).

BM25*e* and TFIDF, which fuse *TF* and the rareness-based indicator *IDF*, can successfully improve *TF*. TFIDF performs better than BM25*e*, which fuses *TF* and *IDF* in a more complicated way (ref. Equation 1). TFIDF performs significantly better than others in Average P@1 and P@2, but not Average P@3 and MAP. On the other hand, ES*e* fuses *TF*, *IDF*, and the concentration-based indicator (*AvgTF*). It performs significantly better than others in Average P@3 and MAP. However, it does not further improve Average P@1 of *AvgTF* (ES*e*: 0.6809 vs. *AvgTF*: 0.6979, ref. Figure 7 and Fig. 1). Therefore, both TFIDF and ES*e* have their weaknesses in CAE identification as well, although they are respectively defined based on the best keyword extractors and article retrievers (ref. Fusion of the indicators).

**Fig. 8:**
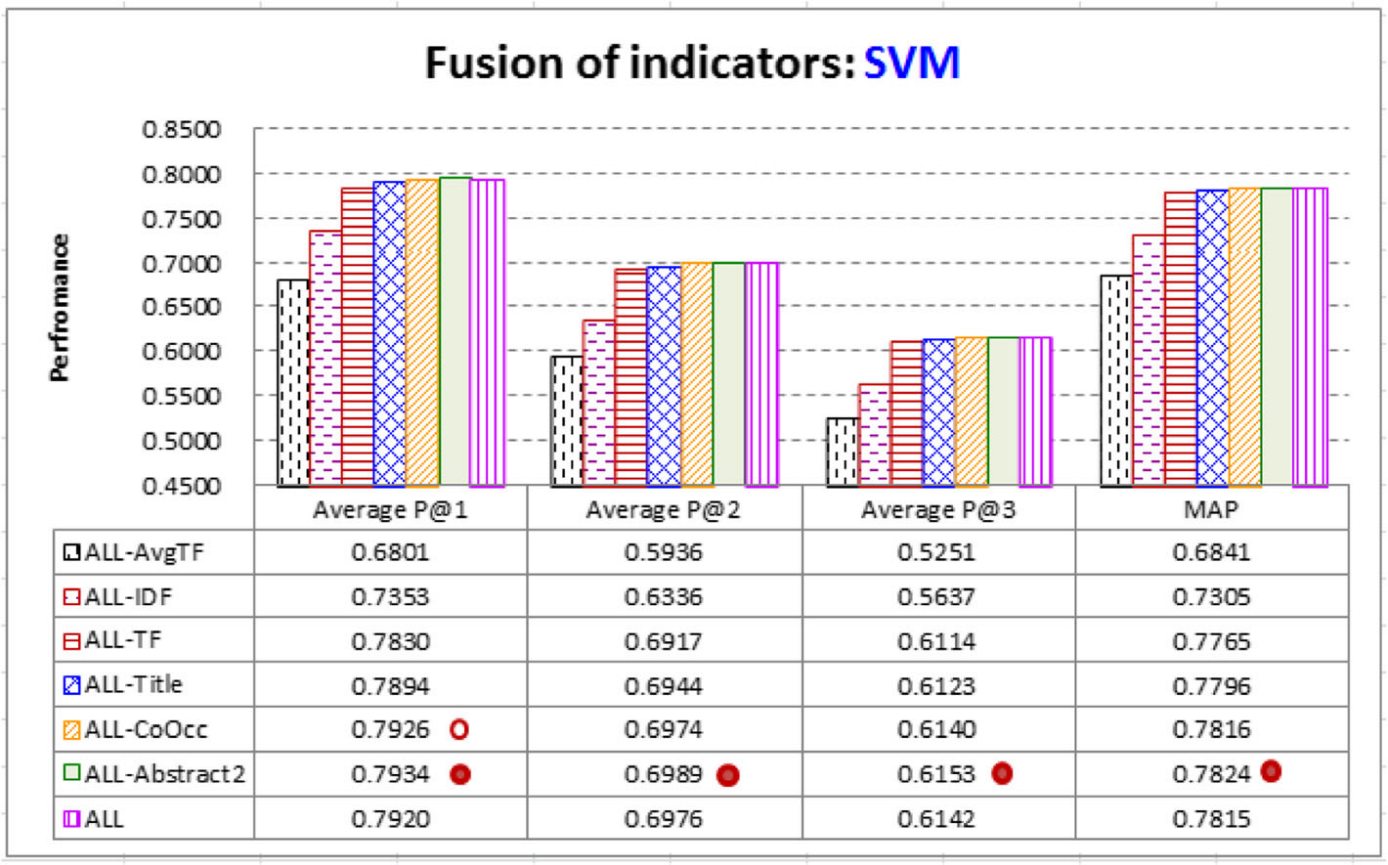
Fusion of indicators by SVM: All the six indicators are fused (see *ALL*), and removal of an indicator *X* from *ALL* is denoted as *ALL*-*X*. We find that removal of a better indicator tends to deteriorate performance more seriously. *ALL-Abstract2* performs significantly better than *ALL* (‘•’ denotes that the indicator performs significantly better than others), indicating that it would be good to integrate all indicators except for *Abstract2*. It performs significantly better than others except for *ALL-CoOcc* on Average P@1 (denoted by ‘ο’). It also performs significantly better than typical fusion strategies (ref. Fig. 7)

It is thus interesting to investigate other ways to fuse the indicators properly. Figure 8 shows the contribution of learning-based fusion by SVM. All the six indicators defined in Table 3 are fused (for the *AbstractX* indicator, we employ *Abstract2*, as it is the best setting for *AbstractX*, ref. Figure 1). Contribution of an indicator to the fused system can be investigated by removing it from the fused system. The results show that removal of a better indicator tends to deteriorate performance more seriously. *ALL-Abstract2*, which fuses all indicators except for *Abstract2*, performs better than all others, including *ALL*, which fuses all the six indicators. Further Removing *CoOcc* from *ALL-Abstract2* gets poorer performance. The performance differences between *ALL-Abstract2* and others are statistically significant, except for *ALL-CoOcc* on Average P@1. Therefore, it may not be necessary to fuse all the six indicators. Without the locality information provided by *Abstract2*, collaboration of the other five indicators has been good in distinguishing CAEs from non-CAEs.

Moreover, as noted above, the two best typical fusion strategies TFIDF and ES*e* have weaknesses. *ALL-Abstract2* tackles the weaknesses by learning-based fusion of five indicators. It performs significantly better than all the typical fusion strategies. There are 9.6% improvement in Average P@1 (0.7934 vs. 0.7239 by TFIDF); 10.9% improvement in Average P@2 (0.6989 vs. 0.6302 by TFIDF); 8.5% improvement in Average P@3 (0.6153 vs. 0.5669 by ES*e*); and 8.3% improvement in MAP (0.7824 vs. 0.7226 by ES*e*).

**Fig. 9:**
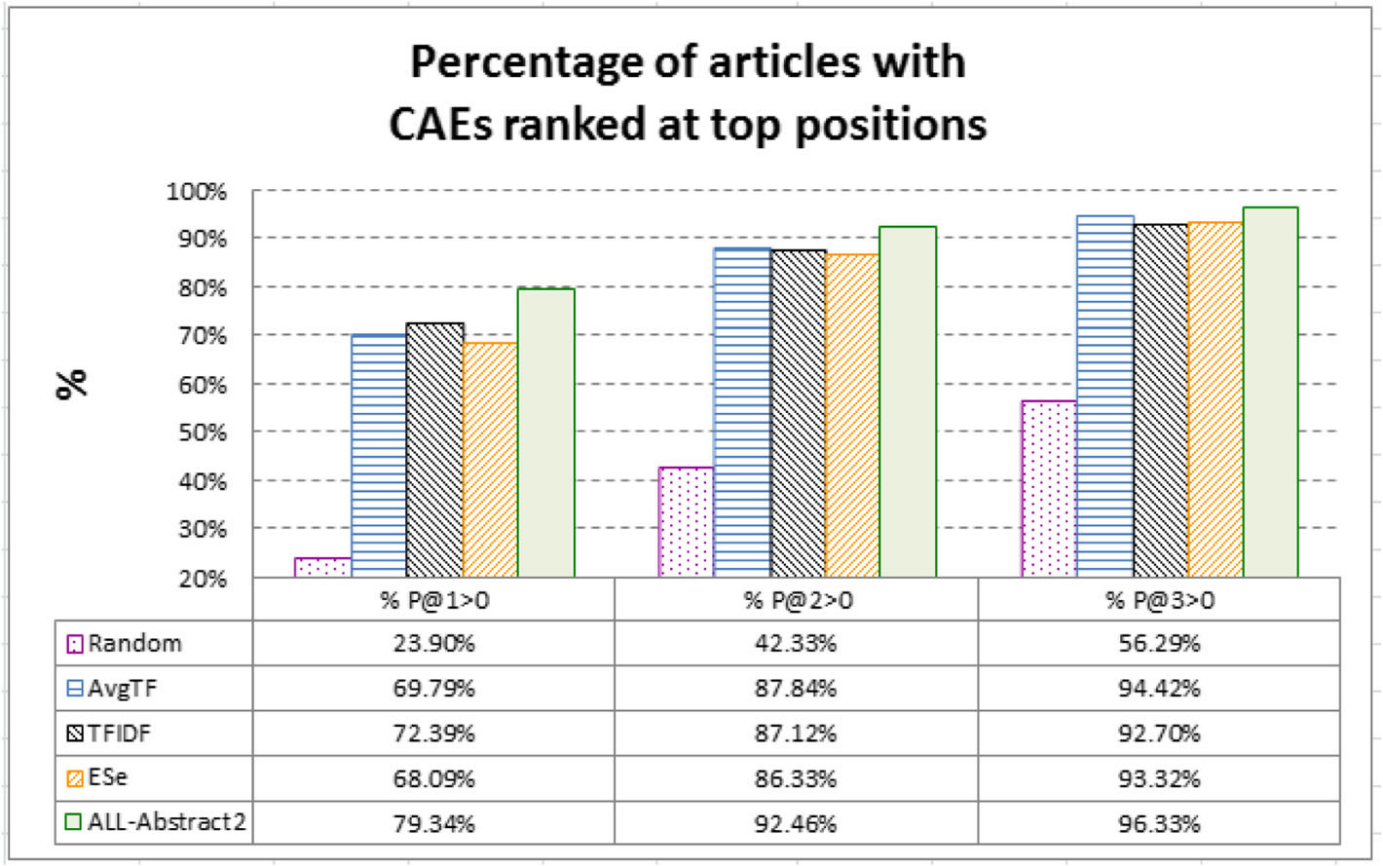
Percentage of articles with CAEs ranked at top positions (i.e., *%P@X > 0*): For 92.46% of the articles, *ALL-Abstract2* ranks at least one of their CAEs at top-2 positions. The percentage achieved by randomly ranking the entities (i.e., the *Random* baseline) is only 42.33%. *ALL-Abstract2* also performs better than the best indicators noted in Figs. 1 and 7 (i.e., *AvgTF*, TFIDF, and ES*e*). It can thus be used to stably identify CAEs for most articles in practice

Figure 9 investigates how CAEs are ranked at top positions for a large percentage of articles (i.e., *%P@X > 0*, ref. Evaluation criteria). For 92.46% of the articles, *ALL-Abstract2* ranks at least one of their CAEs at top-2 positions. The percentage achieved by randomly ranking the entities is only 42.33%. TFIDF and ES*e*, which have better MAP noted above, do not necessarily perform better than the best individual indicator *AvgTF* in *%P@X > 0*, especially when X is 2 and 3. *ALL-Abstract2* performs better than them as well. The results are of practical significance to stable identification of CAEs for most articles.

In conclusion, proper fusion of the indicators is not a trivial task. Typical fusion strategies do not necessarily have better CAE identification performance than individual indicators, even though these fusion strategies were employed by state-of-the-art article retrievers and keyword extractors. Learning-based fusion by SVM is a good way to fuse the indicators. However, it is not necessary to fuse all the indicators. Without the locality information collected from the abstracts of the articles, collaboration of the other indicators has been able to achieve significantly better performance, with most articles (over 92%) having at least one of CAEs successfully ranked at top-2 positions.

#### Case studies

To further investigate potential contributions of the identified CAEs, we conduct case studies to show how the identified CAEs can be visualized to support curation of biomedical databases in practice. Visualization of the CAEs identified from a collection of articles aims at supporting the exploratory analysis of the CAEs. We are motivated by a typical need of biomedical researchers: analysis of a specific research finding is often based on validation of the evidence recently published in multiple articles with focuses on the finding. For example, to curate a gene-disease association, GHR experts need to check multiple articles focusing on the association so that conflicting or unclarified information can be excluded [51]. Therefore, the identified CAEs should be visualized to support the exploration of how *frequently* and *recently* the CAEs are published in articles, as well as how two entities are CAEs in the same articles, which indicates that the two entities may be highly related to each other.

**Fig. 10:**
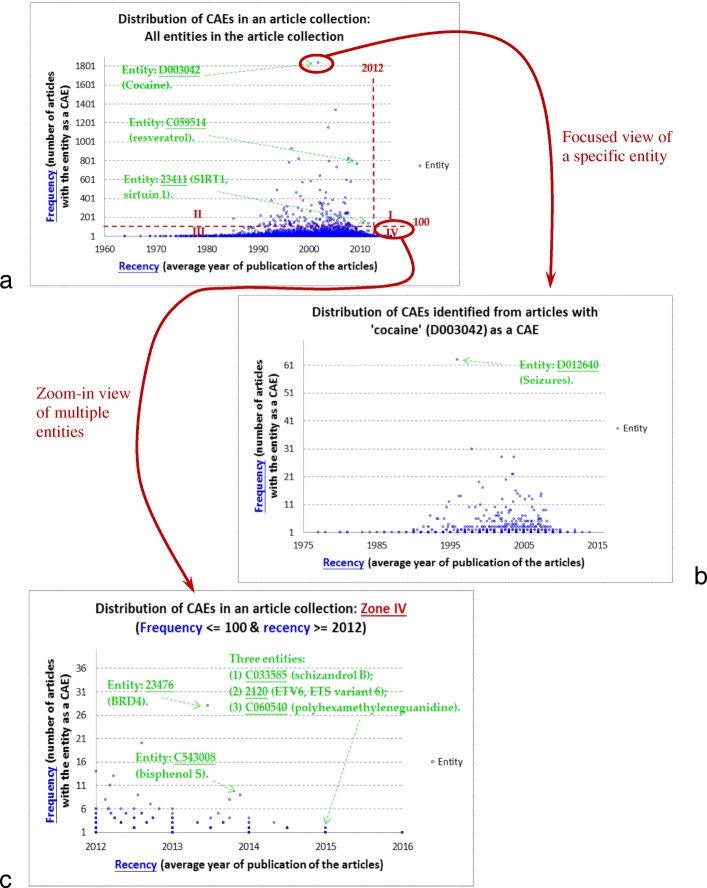
Visualization of the CAEs identified for online exploration: (a) A *frequency-recency* map for the CAEs identified from a collection of articles; (b) *Focused view* of a specific entity of interest (e.g., cocaine, which is a CAE of a large number of articles); (c) *Zoom-in* view of multiple entities (e.g., Zone IV, which contains those entities being studied recently)

More specifically, for each article, top-2 entities identified by *ALL-Abstract2* are treated as CAEs of the article. For each entity *e*, we compute two items: (1) *frequency*: number of articles having *e* as a CAE, and (2) *recency*: average publication year of these articles. A *frequency*-*recency* map can thus be constructed to visualize the CAEs (see Fig. 10a). With the map, researchers can have a global view to navigate on the space of how frequently and recently the CAEs are published in the articles. Consider three CAEs that are published relatively frequently and recently: cocaine (ID in CTD: D003042), resveratrol (ID in CTD: C059514), and SIRT1 (sirtuin 1, ID in CTD: 23411). They are CAEs of 1838, 772, and 135 articles, respectively. To investigate whether the results are helpful for biomedical curators, for each CAE, Eq. 9 is used to measure Jaccard similarity between the sets of articles that are recommended by the system and CTD experts respectively.

9 $$ JaccardSimilarity\left({\mathrm{A}}_1,{\mathrm{A}}_2\right)=\kern0.5em \frac{\mid {\mathrm{A}}_1\cap {\mathrm{A}}_2\mid }{\mid {\mathrm{A}}_1\cup {\mathrm{A}}_2\mid } $$

Jaccard similarities for cocaine, resveratrol, and SIRT1 are 0.8796, 0.875, and 0.8252, respectively. The map can thus serve as a helpful guide to the space of how frequently and recently the CAEs are published in the articles.

Moreover, given the map, two kinds of navigation can be supported: *focused view* of an entity and *zoom-in* view of multiple entities. The focused view is triggered for a specific entity. As a case study, consider a focused view of cocaine (ID in CTD: D003042), which is a CAE with the largest frequency in Fig. 10a. This view focuses on those articles with cocaine as a CAE (see Fig. 10b). It provides a new frequency-recency map to show those entities that are CAEs of those articles with cocaine as a CAE. Therefore, with the focused-view map, researchers can navigate through the information space of how cocaine is related to other entities, as well as those articles that report conclusive findings of both cocaine and the related entities. For example, in Fig. 10b, seizures (ID in CTD: D012640) is an entity with the largest frequency (63 articles), indicating that many articles may have both cocaine and seizures as CAEs, and hence the association between cocaine and seizures deserves investigation. Actually CTD experts used almost all of these articles (61 out of the 63 articles) to curate this association. The focused view can thus support the curation task.

The *zoom-in* view is triggered by selecting a zone in Fig. 10a. There are four zones derived by setting the thresholds for the frequency and the recency. In Fig. 10a, the frequency threshold is set to 100 articles, and the recency threshold to the year of 2012. The four zones aim at supporting different kinds of exploratory analysis. Figure 10c provides a zoom-in view on zone IV, which supports the navigation of those entities that are being studied in *fewer* articles *more recently*. Navigation on this zone can thus facilitate the validation of “emerging” studies on these entities. As case studies, consider three CAEs of multiple articles published most recently. Each of them is CAEs of two articles published in 2015: schizandrol B (entity ID: C033585, article IDs: 25319358, 25,753,323), ETV6 (ETS variant 6, entity ID: 2120, article IDs: 25581430, 25,807,284), and polyhexamethyleneguanidine (entity ID: C060540, article IDs: 25716161, 24,769,016). We find that CTD experts have employed all these articles to curate these CAEs. The zoom-in view can thus be helpful for the curation task as well.

## Discussion

### Application and suggestion

Identification of CAEs can be a new service provided by biomedical search engines (e.g., PubMed), which routinely collect and preprocess articles for subsequent retrieval. For each collected article, the preprocessing process of the search engines can be enhanced by computing the individual and fused indicators for CAE identification. With the CAEs identified for each article, the search engines can facilitate *timely* and *comprehensive* dissemination of conclusive findings in biomedical literature. The new service can also be a good tool for biomedical researchers, curators (e.g., CTD, OMIM, and GHR), and text mining systems that cross-validate conclusive findings on certain entities in multiple articles.

Visualization of CAEs by a frequency-recency map can be a new service provided by biomedical search engines as well. With the new service, researchers can explore the space of CAEs in a collection of articles retrieved for a specific query. The visualization strategy can also be adopted by biomedical databases curated by experts, such as those entity databases that are being maintained by CTD and GHR. By setting a certain condition (e.g., frequency, recency, and entities of interest), researchers can navigate on the space of highly related entities and articles for exploratory analysis.

Another interesting application is the extraction of *key sentences* in biomedical articles. Those sentences that mention CAEs of an article may be the key sentences that describe the main findings of the article. Extraction of the key sentences is thus helpful for the identification and mining of the main findings reported in biomedical literature (e.g., mining associations among entities), which are main goals of many biomedical information extraction and mining systems.

### Limitation and future research

As noted above (ref. The data), for our evaluation purpose, candidate entities in articles are identified based on the vocabulary of CTD, which contains millions of terms for the names, symbols, and synonyms of genes, diseases, and chemicals. The experimental setting provides reliable evidence for performance evaluation, because CTD has employed the vocabulary to curate CAEs in the articles. Other entities not in the vocabulary are not verified by the domain experts of CTD, and hence their effects are not investigated in the paper.

As the CAE identification techniques investigated in this paper work on a given set of candidate entities, they can collaborate with different techniques that map entities in articles to their normalized names or IDs. Previous entity mapping techniques were often developed for specific applications with different performance in different cases. For example, entity recognition techniques were developed for specific domains or types of entities, such as chemicals [52], genes [53], and diseases [54]. Mapping the entities into suitable IDs is an important research topic as well (e.g., mapping of genes [55]) for which tools were implemented (e.g., MetaMap, available at https://metamap.nlm.nih.gov/) and techniques were developed with different performance in different cases [56]. By collaborating with those entity mapping techniques that are tuned for specific applications, CAE identification may be improved for the applications.

CAE identification may also be improved by collecting more information from multiple articles, based on three observations: (1) given two entities *e*_1_ and *e*_2_ that are CAEs in an article, there may be an association <*e*_1_, *e*_2_ > between them, (2) associations between CAEs may be used to infer possible associations (e.g., given <*e*_1_, *e*_2_ > and < *e*_2_, *e*_3_>, an inferred association may be <*e*_1_, *e*_3_>), and (3) if two candidate entities in an article *a* are involved in an inferred association (e.g., *e*_1_ and *e*_3_ are candidate entities in *a*, and < *e*_1_, *e*_3_ > is an inferred association), they are likely to be CAEs of *a*. Therefore, CAE identification for an article may be improved by *CAE-based association mining* on a collection of articles. The CAE identification techniques investigated in this paper can be used to identify CAE associations (based on the 1st observation). Novel techniques may be developed to infer possible associations and refine CAE identification for each article (based on the 2nd and 3rd observations, respectively).

The CAE visualization strategy noted above (ref. Case studies) can be extended as well. An interesting extension is network-based navigation of conclusive findings on a set of entities of interest. Given a set *E*_*i*_ of entities of interest, the system identifies a set *E*_*h*_ of entities that are *highly related* to the entities in *E*_*i*_. Two entities are highly related if they are CAEs of the same article (i.e., the article reports conclusive findings on them). The system then visualizes *E*_*i*_ and *E*_*h*_ with an association network in which a node is an entity, and an edge between two nodes indicates that they are highly related. The users can click on any edge between two entities to check the distribution of those articles that have the two entities as CAEs. With the CAE network, biomedical researchers can have global and detailed views on a set of entities among which associations are reported as conclusive findings in literature.

## Conclusion

CAEs in a biomedical article *a* are specific entities on which conclusive associations are reported in *a*. They are different from keywords (e.g., MeSH terms) employed to index (classify or label) *a*. This paper is the first study to investigate how five types of statistical indicators can contribute to prioritizing candidate entities in the title and the abstract of an article so that CAEs can be ranked on the top for exploratory analysis.

The results show that these indicators have significantly different performance. Some indicators do not perform well in CAE identification, even though they were used in many article retrievers and keyword extractors. Learning-based fusion of certain indicators can successfully rank CAEs in most articles at top-2 positions. As it can work on titles and abstracts of articles, which are more commonly available than full texts of the articles, it can be applicable to much more articles. By visualizing the identified CAEs with frequency-recency maps, biomedical researchers can navigate to check how frequently and recently the CAEs are published in articles, as well as how two entities are CAEs in the same articles (i.e., they may be highly related to each other).

The results are of both technical and practical significance to the indexing of biomedical articles to support validation of highly related conclusive findings in biomedical literature. They can also be used to enhance biomedical search engines, curated databases, and text mining systems, which often serve as essential components of many biomedical information processing systems.

## Additional file

Additional file 1: Biomedical articles that are employed as the experimental data. There are 60,507 articles, which amount to about 50% of the articles in CTD. Each article has a PubMed ID, followed by its CAEs (represented by their IDs in CTD and separated by ‘|’). CAEs of an article *a* are the specific entities involved in the associations that CTD experts curated based on the conclusive findings of *a*. (TXT 4849 kb)

## Abbreviations

%P@X > 0: Percentage of articles having at least one CFE ranked at top-X positions
Average P@X: Average precision at top-X
CAE: Conclusive association entity
IDF: Inverse document frequency
MAP: Mean average precision
ProbGain: Probability gain
SVM: Support vector machine
TF: Term frequency
